# Supplementary material for: Oxidative Stress, Cytotoxic and Inflammatory Effects of Urban Ultrafine Road-Deposited Dust from the UK and Mexico in Human Epithelial Lung (Calu-3) Cells
Source: Antioxidants (Basel). 2022 Sep 14;11(9):1814. doi: 10.3390/antiox11091814 (PMC9495992; doi:10.3390/antiox11091814)
Supplement: Supplementary file 1 [file antioxidants-11-01814-s001.zip › antioxidants-1853651-supplementary.pdf]

Supplemental Material

Oxidative stress, cytotoxic and inflammatory effects of urban ultrafine road-deposited dust from the UK and Mexico in human epithelial lung (Calu-3) cells

Jessica Hammond, Barbara A. Maher, Tomasz Gonet, Francisco Bautista, and David Allsop.

Table of Contents

Magnetic properties of the total and ultrafine (< 220 nm) road-deposited dust particles

Table S1. Overview of cytotoxicity studies of road-deposited dust and roadside airborne PM in human and rodent models.

Table S2. Elemental composition of the analysed UF-RDPs from Lancaster, Birmingham, and Mexico City

Table S3. Endotoxin content of ultrafine road-deposited dust particles.

Figure S1. Isothermal remanence magnetisation (IRM) for the bulk (unfiltered) PM from the three studied sampling sites

Figure S2. Zero-field changes in IRM during cooling of the Mexican UF-RDPs, after acquisition of IRM (at 1T) at 300 K.

Figure S3. Release of pro-inflammatory cytokines (ng/ml) in Calu-3 cells treated with ultrafine road-deposited dust particles

Figure S4. MTS-adjusted release of pro-inflammatory cytokines (ng/ml) in Calu-3 cells treated with ultrafine road-deposited dust particles

Figure S5. Oxidative stress in Calu-3 cells induced by ultrafine road-deposited dust particles

Figure S6. Release of pro-inflammatory cytokines in Calu-3 cells treated with ultrafine road-deposited dust particles

References

Magnetic properties of the total and ultrafine (< 220 nm) road-deposited dust particles

Isothermal remanent magnetisation (IRM) is a commonly used parameter in environmental magnetism. It is dependent on the concentration, mineralogy, and grain size distribution of the magnetically ordered grains within a sample. This parameter is especially sensitive to the presence of strongly magnetic Fe forms, including metallic Fe (α-Fe), magnetite (Fe_3O_4), and its oxidised counterpart, maghemite (γ-Fe_2O_3).

Low-temperature magnetic measurements can be used to identify certain Fe forms in a sample. For instance, if magnetite is present in the sample, the Verwey transition is visible in the zero-field cooling/heating curves [75]. This first order transition is displayed by a sudden decrease in IRM at ~70 – 130 K. The Verwey transition is usually sharp for pure magnetite while partially oxidised (maghemitised) magnetites usually display wider and indistinct Verwey transitions [74]. The Verwey transition is also broadened for magnetites ~20-30 nm in size, close to the border of stable single domain/ superparamagnetic grains [74].

Supplementary Figure S1 shows the room-temperature (300 K) IRM for bulk, unfiltered road-deposited PM samples from the three locations. The Birmingham sample displays the highest IRM, reaching $71 \cdot 10^{-3}$ Am^2/kg, followed by Mexico City ($51 \cdot 10^{-3}$ Am^2/kg), and Lancaster ($36 \cdot 10^{-3}$ Am^2/kg) (Supplementary Figure S1; Supplementary Table S2). These IRMs correspond to approximately ~0.32 – 0.95 wt.% of magnetite for Birmingham, ~0.24 – 0.79 wt.% of magnetite for Mexico City, and ~0.18 – 0.63 wt.% of magnetite in Lancaster.

The Verwey transition, indicative of the presence of magnetite, is visible on the heating curve at ~75 – 90 K, measured for ultrafine road-deposited dust particles (UF-RDPs) from Mexico City (Supplementary Figure S2). This transition is broad and indistinct, probably reflecting very small grain size (~20 – 30 nm) and/or partial oxidation (maghemitisation) of the magnetite grains [74]. The presence of superparamagnetic (< ~30 nm) grains is further supported by a relatively low temperature of the Verwey transition and ~43% increase in IRM at low-temperature (10 K), compared to the IRM measured at room temperature; and ~16% increase in IRM at 10 K, compared to IRM at 50 K [74].

Supplementary Table S1. Overview of selected cytotoxicity studies of road-deposited dust and roadside airborne PM in human and rodent models.

Size	Method of collection	Location	Cell line/model	Exposure	Dose	Assays/outcomes	Ref.
Sieved to <75 µm, then aerosolized to collect $PM_{2.5}$ and PM_{10}.<br>(Settled dust)	Road vacuum sweeper	Gangdong-Gu, Seoul, Korea	Human corneal epithelial cells (2.040 pRSV-T)	Liquid culture	10 - 500 µg/ml	MTT (cell viability) – ↓<br>LDH (cytotoxicity) – ↑<br>NO (Oxidative stress) – ↑ (500µg/ml total $PM_{2.5}$ and PM_{10})<br>IL-8 (cytokine release) -↑ (total $PM_{2.5}$ and PM_{10})<br>ROS – ↑ (more so for $PM_{2.5}$ than PM_{10})	[59]
Sieved to <100 µm<br>(Settled dust)	Vacuum cleaner	Daejeon, South Korea	Human adherent lung fibroblasts (WI-38) and human foreskin fibroblasts (BJ)	Liquid culture	0.01 - 2 mg/ml	WST8 (cell viability) – ↓<br>Association with metals and decrease in viability	[56]
Sieved to <100 µm<br>(Settled dust)	Vacuum cleaner	South Korea	Human adherent lung fibroblasts (WI-38) and human foreskin fibroblasts (BJ)	Liquid culture	0.01 - 2 mg/ml	CCK8 (cell viability) – ↓<br>Different responses 28 different samples<br>PAH and metal content correlated to cytotoxicity	[18]
Sieved to <100 µm. Extracts were filtered with 0.2 µm syringe filter<br>(Settled dust)	Sweeping	Guangzhou, China	Human hepatocellular liver carcinoma (HepG2) and human skin derived keratinocyte (KERTr)	Liquid culture	334 and 667 µg/ml	MTT (cell viability) – ↓	[54]
$PM_{2.5}$<br>(Settled dust/Airborne PM)	Sweeping to simulate road cleaning then samples collected by pumping air	Harbin, Beijing, Tianjin, Lanzhou, Xi'an, Nanjing, Shanghai, Chengdu, Wuhan, and Guangzhou, China	Human pharynx epithelial cells (FaDu)	Liquid culture	50 µg/ml	SRB (viability) – ↓<br>LDH (cytotoxicity) – ↑<br>IL-6 (cytokine release) - ↑<br>HMBG1 (protein) - ↑<br>RAGE (protein) - ↑<br>Occludin (protein) - ↓<br>E-cadherin (protein) - ↓<br>EGFR (protein) - ↑<br>p-EGFR (protein) - ↑	[58]
$PM_{2.5}$<br>(Settled dust/Airborne PM)	Sweeping to simulate road cleaning then samples collected by pumping air	Harbin, Beijing, Tianjin, Lanzhou, Xi'an, Nanjing, Shanghai, Chengdu, Wuhan, and Guangzhou, China	Human alveolar epithelial cells (A549)	Liquid culture	50 µg/ml	ROS – ↑<br>LDH (cytotoxicity) – ↑ (in industrial cities)<br>Heavy metals correlated to ROS<br>LDH correlated to organic compounds and cations<br>LDH production associated with ROS<br>Vehicle emissions and coal combustion were strongly correlated with cellular ROS	[55]

<38 µm then aerosolized in $PM_{2.5}$ filter sampling system (Settled dust)	No data	Urban Gwangju, South Korea	Human alveolar epithelial cells (A549)	Liquid culture	0 - 150 µg/ml	DCFDA (ROS) – ↑	[57]
nPM (<200nm) (Airborne PM)	High volume ultrafine particle sampler	Near CA-110 freeway, Los Angeles, California, USA	Hippocampal slices<br>Primary neurons<br>Primary glial cultures	Liquid culture	1 - 20 µg/ml	Cytotoxicity (LDH) – ↑<br>Neurite outgrowth – ↓ (inhibition)<br>PI uptake – ↑ (neurotoxic)<br>IL-1α (cytokine mRNA) – ↑<br>TNFα (cytokine mRNA)– ↑<br>IL-1β (cytokine mRNA) – ↑<br>IL-6(cytokine mRNA) – ↑	[60]
nPM (<200nm) (Airborne PM)	High volume ultrafine particle sampler	Near CA-110 freeway, Los Angeles, California, USA	Primary mixed glia (microglia and astrocytes)	Liquid culture	10 µg/ml	TNFα (cytokine release) – ↑<br>IL-1β (cytokine release) – ↑<br>IL-6(cytokine release) – ↑<br>KC (cytokine release) – ↑<br>IFN-γ (cytokine release) – ↑<br>IL-5 (cytokine release) – ↑<br>IL-4(cytokine release) – ↓	[61]

Supplementary Table S2. Elemental composition of the analysed UF-RDPs from Lancaster, Birmingham, and Mexico City (b.d.l. = below detection limit).

Element	Method	Lancaster		Birmingham		Mexico City	
		Concentration	St.dev [%]	Concentration	St.dev [%]	Concentration	St.dev [%]
Al (ppm)	ICP-OES	57.9	2.3	55.8	1.8	97.1	3.2
Ba (ppm)	ICP-MS	0.4	0.6	0.5	6.5	1.6	2.5
Ca (ppm)	ICP-OES	770	0.5	743	0.9	3651	0.7
Cd (ppm)	ICP-OES	1.1	13.9	6.1	4.2	b.d.l.	-
Co (ppm)	ICP-MS	0.4	1.3	0.7	50.7	2.8	2.8
Cr (ppm)	ICP-MS	18.7	1.0	15.0	2.6	125.3	0.6
Cu (ppm)	ICP-MS	29.1	1.7	19.7	0.9	67.2	0.3
Fe (ppm)	ICP-OES	55.9	1.4	14.7	3.3	77.1	1.3
K (ppm)	ICP-OES	256.8	0.8	4.6	5.7	789.7	0.6
Mg (ppm)	ICP-OES	92	3.2	95	3.5	736	1.8
Mn (ppm)	ICP-OES	5.3	1.2	2.5	2.4	8.1	1.9
Na (ppm)	ICP-OES	105,489	0.6	1,003	10.5	42,163	1.0
Ni (ppm)	ICP-OES	b.d.l.	-	1.1	14.9	8.3	12.0
P (ppm)	ICP-OES	43.4	6.1	20.8	20.3	127.9	10.2
Pb (ppm)	ICP-MS	8.0	2.2	0.7	39.8	1.2	6.5
Si (ppm)	ICP-MS	0.08	2.5	0.02	15.5	0.06	2.1
Sn (ppm)	ICP-MS	1.6	2.0	1.7	12.6	15.6	1.7
Sr (ppm)	ICP-OES	0.8	1.9	0.1	6.4	2.1	1.1
Ti (ppm)	ICP-OES	5.3	2.3	2.1	2.1	12.1	1.6
V (ppm)	ICP-MS	5.3	0.2	2.0	18.1	11.1	1.9
Zn (ppm)	ICP-MS	374	2.7	277	5.7	1337	1.2
Ce (ppb)	ICP-MS	3.0	26.6	3.2	17.8	3.1	35.0
Er (ppb)	ICP-MS	0.2	109.1	0.3	42.3	0.4	136.6
Hg (ppb)	ICP-MS	8.6	20.1	6.1	24.0	8.1	17.8
Mo (ppb)	ICP-MS	0.01	32.1	0.01	30.8	0.01	49.2
Zr (ppb)	ICP-MS	0.5	51.4	0.4	80.3	0.8	26.3

Supplementary Table S3. Endotoxin content of ultrafine road-deposited dust particles.

City	Endotoxin (EU/mg)
Lancaster	9.25
Birmingham	9.80
Mexico City	8.75

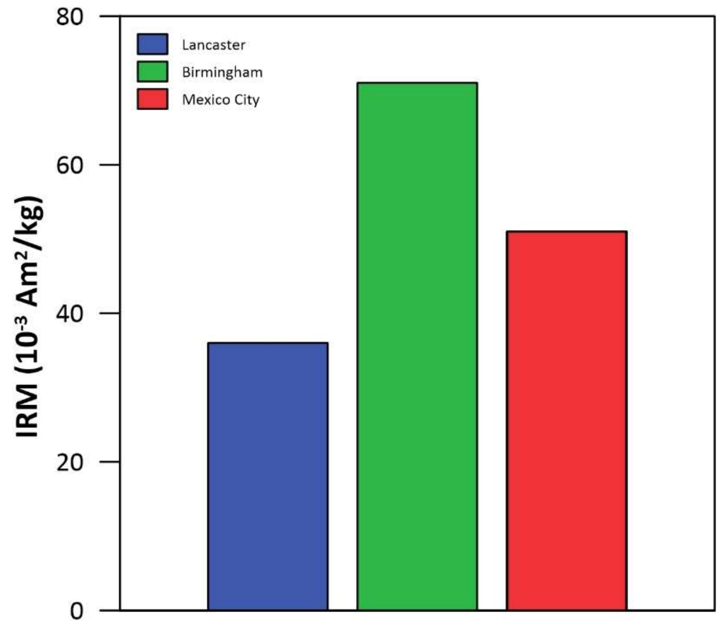

Supplementary Figure S1. Isothermal remanence magnetisation (IRM) for the bulk (unfiltered) RD from the three studied sampling sites.

Note. The IRM of bovine serum albumin (BSA) was also measured and was several orders of magnitude lower than the bulk RD samples (IRM 0.0014 x 10^{-3} Am^2/kg).

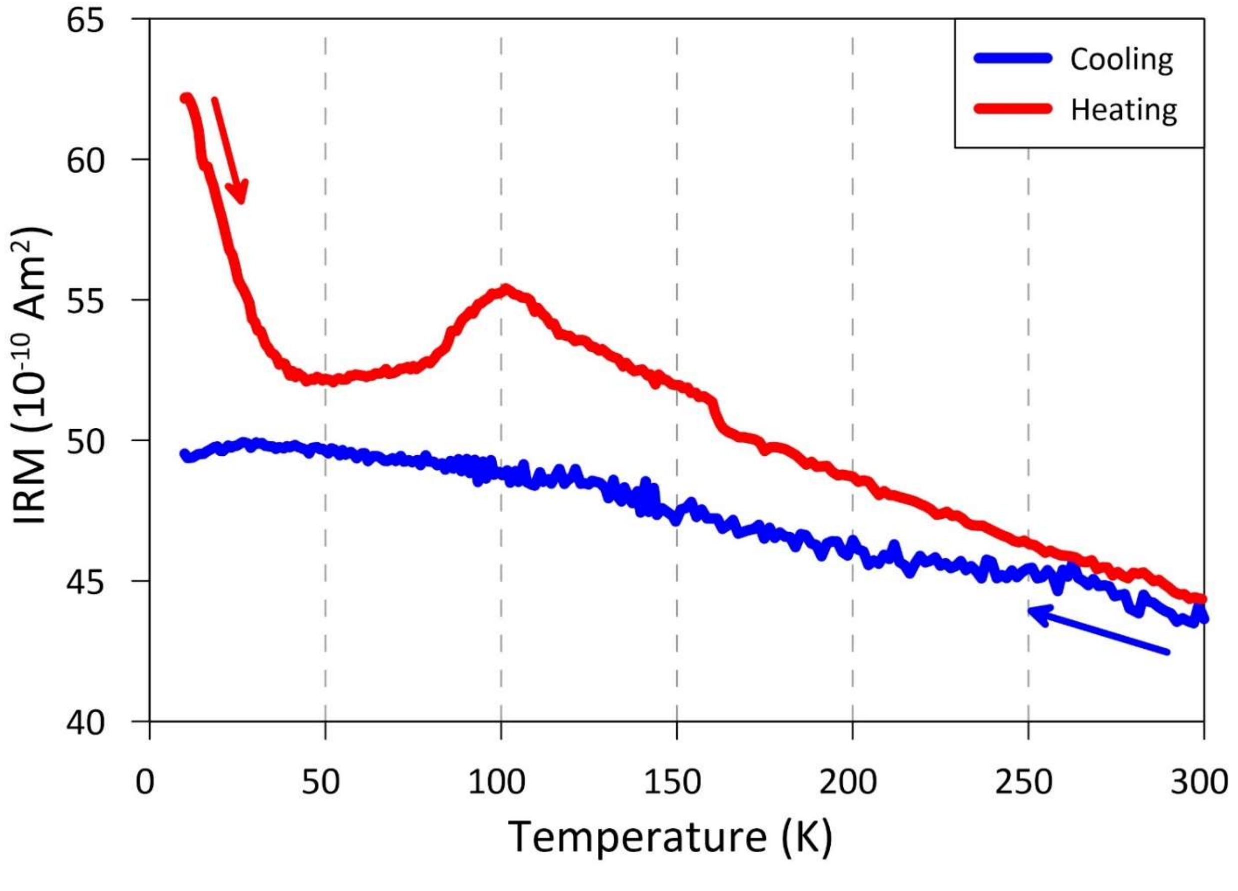

Supplementary Figure S2. Zero-field changes in IRM during cooling of the Mexican UF-RDPs, after acquisition of IRM (at 1 T) at 300 K. The Verwey transition at ~75 – 100 K identifies the presence of magnetite. The increase in IRM at low temperatures (~43% increase in IRM at 10 K compared to room temperature IRM; ~16% increase in IRM at 10K compared to IRM at 50 K) indicates the presence of superparamagnetic grains (~20 – 30 nm).

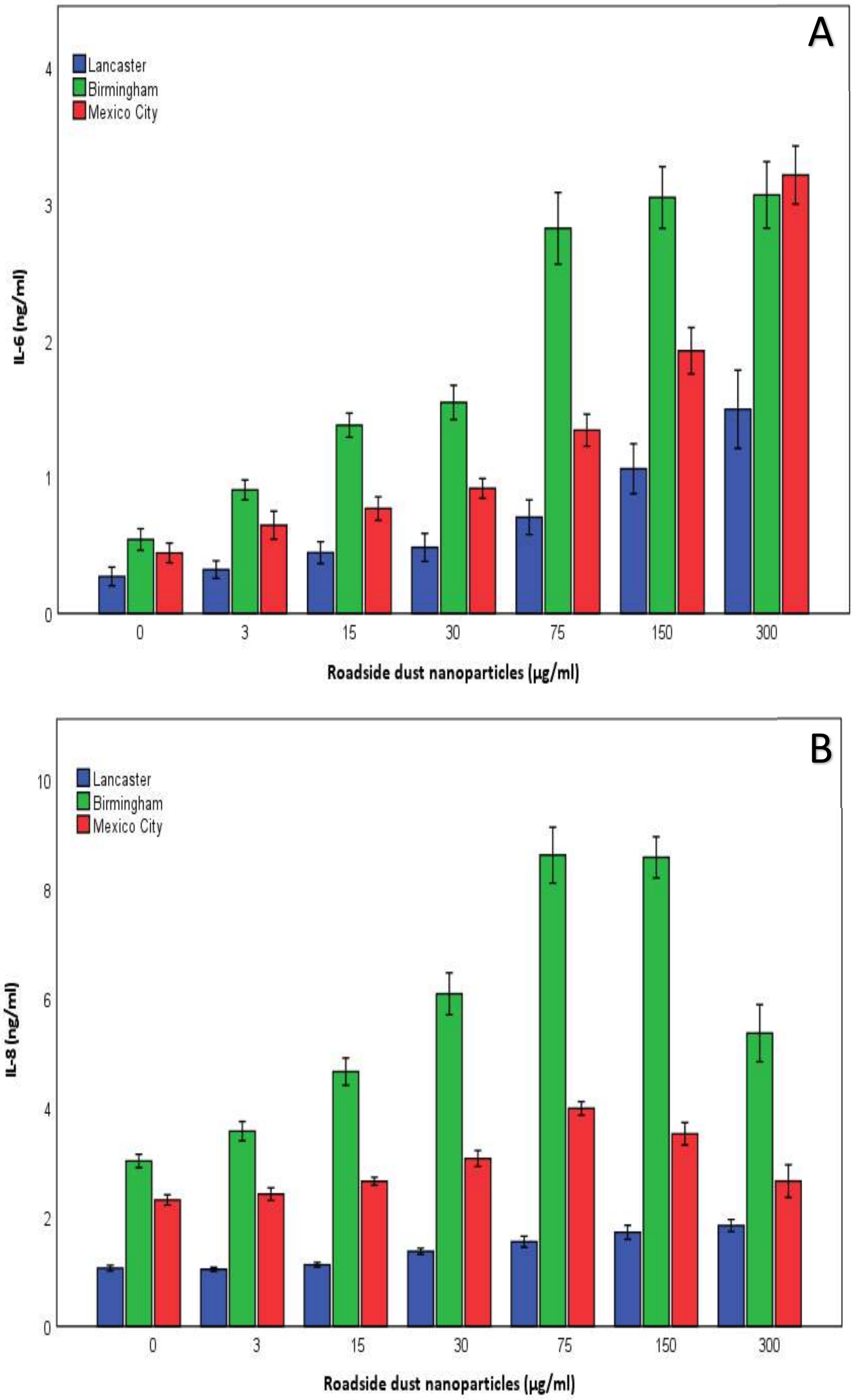

Supplementary Figure S3. Release of pro-inflammatory cytokines (ng/ml) in Calu-3 cells treated with <220 nm road-deposited dust particles. Calu-3 cells were exposed (at doses from 3 to 300 µg/ml) to the sub-micrometre-sized fraction of road-deposited dust particles from Lancaster (UK), Birmingham (UK) and Mexico City (Mexico) for 24 h and the media collected. The collected media samples were quantified for IL-6 (A) and IL-8 (B) concentrations via ELISA.

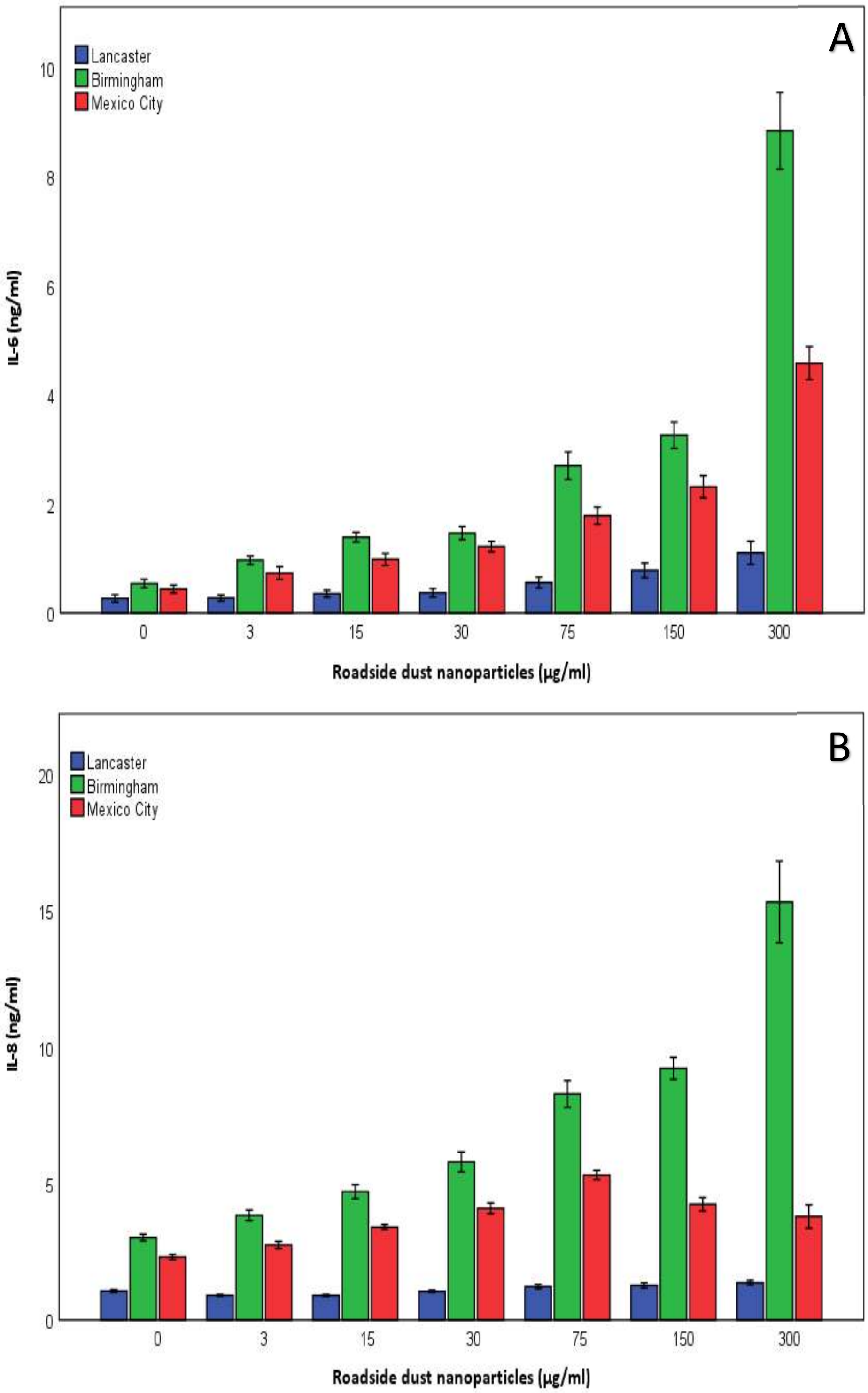

Supplementary Figure S4. MTS-adjusted release of pro-inflammatory cytokines (ng/ml) in Calu-3 cells treated with <220 nm road-deposited dust particles. Calu-3 cells were exposed (at doses from 3 to 300 µg/ml) to the sub-micrometre-sized fraction of road-deposited dust particles from Lancaster (UK), Birmingham (UK) and Mexico City (Mexico) for 24 h and the media collected. The collected media samples were quantified for IL-6 (A) and IL-8 (B) concentrations via ELISA. Data were adjusted for potential change in cell number using MTS assay data.

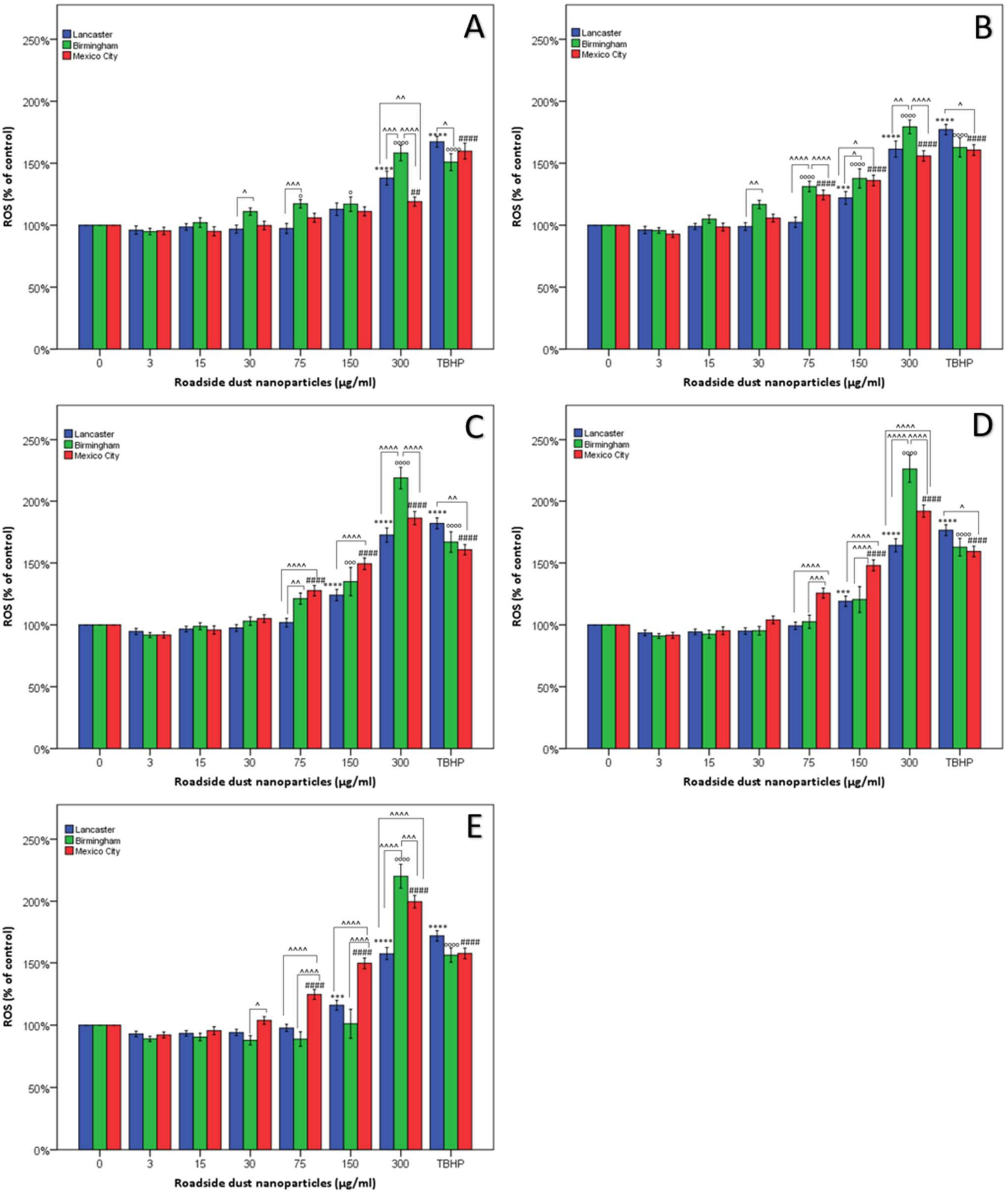

Supplementary Figure S5. Oxidative stress in Calu-3 cells induced by < 220 nm road-deposited dust particles. Calu-3 cells were loaded with the ROS probe CM DCFH-DA and exposed to the sub-micrometre-sized fraction of road-deposited dust particles (at doses from 3-300 µg/ml) from Lancaster (UK), Birmingham (UK) and Mexico City (Mexico). Generation of fluorescence (indicative of ROS generation) was measured after 0.5H (A), 1H (B), 2H(C), 3H (D) and 4H (E). Tert-butyl hydroperoxide (TBHP) was used at 100 µM as a positive control. Statistical analysis was conducted via one-way ANOVA with Dunnett's post-hoc, comparing treated cells to the untreated control. (* Lancaster, $_{0}$ Birmingham, # Mexico City) and two-way ANOVA with Bonferroni correction in order to compare the impacts of the UF-RDPs from the three different locations (^).

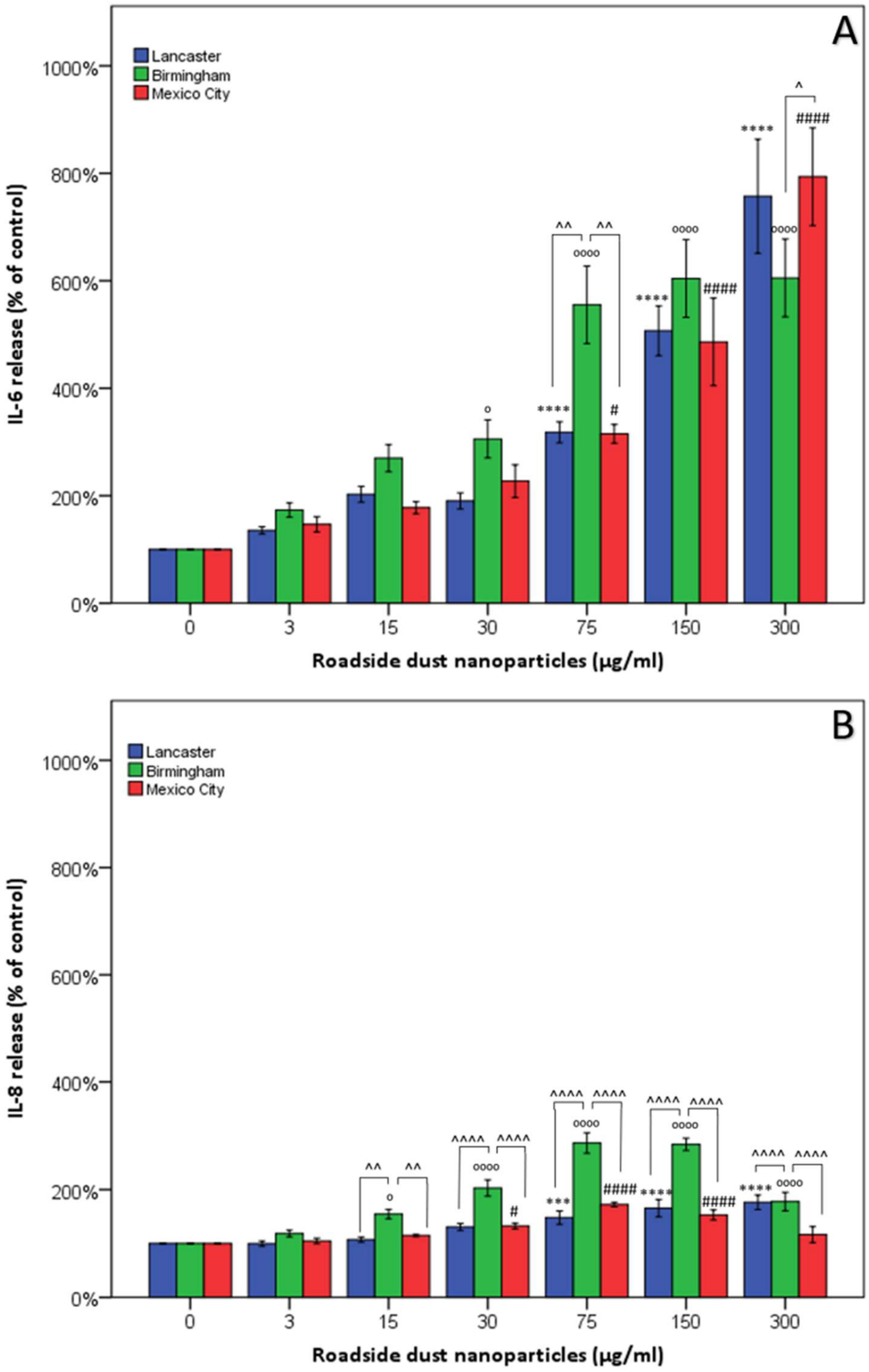

Supplementary Figure S6. Release of pro-inflammatory cytokines in Calu-3 cells treated with <220 nm road-deposited dust particles. Calu-3 cells were exposed (at doses from 3 to 300 μg/ml) to the sub-micrometre-sized fraction of road-deposited dust particles from Lancaster (UK), Birmingham (UK) and Mexico City (Mexico) for 24 h and the media collected. The collected media samples were quantified for IL-6 (A) and IL-8 (B) concentrations via ELISA. Statistical analysis was conducted via one-way ANOVA with Dunnett's post-hoc, comparing treated cells to the untreated control. (* Lancaster, $_0$ Birmingham, # Mexico City) and two-way ANOVA with Bonferroni correction in order to compare the impacts of the UF-RDPs from the three different locations (^).
